# Supplementary material for: Lysine demethylase 7a regulates murine anterior-posterior development by modulating the transcription of Hox gene cluster
Source: Commun Biol. 2020 Nov 30;3:725. doi: 10.1038/s42003-020-01456-5 (PMC7704666; doi:10.1038/s42003-020-01456-5)
Supplement: Supplementary file 1 — Supplementary Information [file 42003_2020_1456_MOESM1_ESM.pdf]

Supplementary Table 1: List of primers and oligonucleotides used for construct generation.

	Sequence
Kdm7a_51+_for_pCAG-EGxxFP	AGCTACATAATGAAACCATGTCTCC
Kdm7a_564-_for_pCAG-EGxxFP	GTACCAAGGCAGAAAATGTTTAAGA
sgRNA_867F_for_pX330	CACCGTATAGCCAGAAAACTTTCA
sgRNA_867R_for_pX330	AAACTGAAAGTTTTCTGGCTATAC
sgRNA_868F_for_pX330	CACCGATAGCCAGAAAACTTTCAT
sgRNA_868R_for_pX330	AAACATGAAAGTTTTCTGGCTATC
sgRNA_868F_for_DR274	TAGGTATAGCCAGAAAACTTTCAT
sgRNA_868R_for_DR274	AAACATGAAAGTTTTCTGGCTATC

Supplementary Table 2: Primers used for mRNA quantification

Target gene	Strand	Sequence (5′ end)
Hoxa1	Forward	CTCCCAAAACAGGGAAAGTTGGA
	Reverse	TTGAAGTGGAACTCCTTCTCCAG
Hoxb1	Forward	CCCACCTAAGACAGCGAAGG
	Reverse	TGGTGAAGTTTGTGCGGAGA
Hoxd1	Forward	CCCCCAAGAAAAGCAAACTGTC
	Reverse	GGTTCTGGAACCAGATTTTGACC
Hoxa2	Forward	CCACAAAGAATCCCTGGAAATAGC
	Reverse	TCACTTGTCTCTCGGTCAAATCC
Hoxb2	Forward	GCGAAATTGCTCCATTGCATAAAC
	Reverse	ACCCAATCTCCCTCTCAAATTCAA
Hoxa3	Forward	TTAGGTCCAGAAGTGTCCAAACC
	Reverse	CAGTGTCCAGGCACTCTTAACAT
Hoxb3	Forward	AAAAAGTGTTAGCCGTCTCTCCG
	Reverse	CGAGAAATCTCCCCTCCTCTGA
Hoxd3	Forward	TTCCACTTCAACCGCTATCTGTG
	Reverse	GGAGAATGCAGGATGCCCTTAG
Hoxa4	Forward	GAGCGCCGTCAACTCCAGTTAT
	Reverse	AGTGGAATTCCTTCTCCAGTTCC
Hoxb4	Forward	GAGCACGGTAAACCCCAATTACG
	Reverse	GAAACTCCTTCTCCAACTCCAGG
Hoxc4	Forward	AGCACGGTGAACCCCAATTA
	Reverse	CGATCTCGATCCTTCTCCTTCG
Hoxd4	Forward	TGTAGCGAGCAGCAATACTTAGA
	Reverse	AGTGAATTCTCCTACAAGCCTGG
Hoxa5	Forward	AGTGAATTCTCCTACAAGCCTGG
	Reverse	AGTGGAATTCTTTCTCCAGCTCC
Hoxb5	Forward	CTTCACATCAGCCACGATATGAC
	Reverse	GGAACCAGATTTTGATCTGACGC
Hoxc5	Forward	ACATGAGCCACGAGACGGAT
	Reverse	ATTCTTTCTCGAGTTCCAGGGTC
Hoxa6	Forward	GGGACTACCTGCACTTTTCTCC
	Reverse	ATACACGGCACCCGCACAG
Hoxb6	Forward	TGAATTCGTGCAACAGTTCCTCT
	Reverse	CCGGTTCTGAAACCAAATCTTGA
Hoxc6	Forward	GAATGAATTCGCACAGTGGGGTC
	Reverse	GAGTTAGGTAGCGGTTGAAGTGA
Hoxa7	Forward	AGTTCAGGACCCGACAGGAAG
	Reverse	TGGAATTCCTTCTCCAGTTCCAG

Hoxb7	Forward	TCAAGGAATCTCGTAAAACCGAC
	Reverse	TGAACTCATAATTTGGCCGGATG
Hoxb8	Forward	GGACCTTTTAAAACTCGGTGCAA
	Reverse	TCTTTCTAAATGTCAGGGTCGCT
Hoxc8	Forward	GGATGAGACCCCACGCTCC
	Reverse	CTTGTCTTTCTGTCAGTCCCAGG
Hoxd8	Forward	CCGCGAAGTTTTACGGATACGAT
	Reverse	GGAGCTGCTTGTGGTCTCATC
Hoxa9	Forward	GCATTAAACCTGAACCGCTCTC
	Reverse	CGGGTTATTGGGATCGATGGG
Hoxb9	Forward	AAAGAGAGGCCGGATCAAACCAA
	Reverse	GGTCCCTGGTGAGGTACATATTG
Hoxc9	Forward	AAAAGATCAGAGACTGCAGGAGC
	Reverse	GATGAAAATGCCAGTCCCAGAAG
Hoxd9	Forward	CAACTTGACCCAAACAACCCTG
	Reverse	CTCTAGCGTCTGGTATTTGGTGT
Hoxa10	Forward	GAGTCCTAGACTCCACGCCA
	Reverse	CCTTTGGAACTGCCCAGGGA
Hoxc10	Forward	CGGATAACGAAGCGAAAGAGGAG
	Reverse	AATGGTCTTGCTAATCTCCAGGC
Hoxd10	Forward	CAGGAGAAGGAAAGCAAAGAGGA
	Reverse	GGTGAGGTTAACGCTCTTACTGA
Hoxa11	Forward	ATATCATCCCACCACTGATCTGC
	Reverse	CACAGCCTCTGGAGTTTTCAATG
Hoxc11	Forward	ATGTTTAACTCGGTCAACCTGGG
	Reverse	TAAGTGCAACTGGGCAGATAGAG
Hoxd11	Forward	GGCGAGATCTGTAGGAAGTTAGG
	Reverse	CCCAAAGGTACATTTCCCAGAGT
Hoxc12	Forward	CCTACTCAACGAGGGCAATAAGA
	Reverse	TGATGAACTCGTTGACCAGAAAC
Hoxd12	Forward	CCAACCTTTAGCAAGATGCACAA
	Reverse	ACATAAACGGCAACTGTTAGCAC
Hoxa13	Forward	GAAAGAACTCGAACGGGAATACG
	Reverse	CTCCTGTTCTGGAACCAGATTGT
Hoxc13	Forward	CAGTCAGGTGTACTGCTCCAAG
	Reverse	TCTTTGGTGATGAATTTGCTGGC
Hoxd13	Forward	TCCTTTCCAGGAGATGTGGCT
	Reverse	TCTCTCCGAAAGGTTCGTGG
Kdm7a	Forward	AGCAATAGAGGAGGAAAATGGCA
	Reverse	CAAGGTTAGAAGGAGTTCGGACA

Supplementary Table 3: Primers used for ChIP followed by real-time PCR

	Strand	Sequence (5′ end)
Actb	Forward	GCCGTATTAGGTCCATCTTGAGA
	Reverse	CAAACCGGTTTGGACAAAGACC
Hoxa3	Forward	TCGTGCTGCTAAATATTGCTGAC
	Reverse	GCGCAAATCCATCTTACTCTCAA
Hoxa13	Forward	TCCCTAAAACATGCCAGGACATC
	Reverse	AGTCAGGTAAATTCTCCAGTGGC

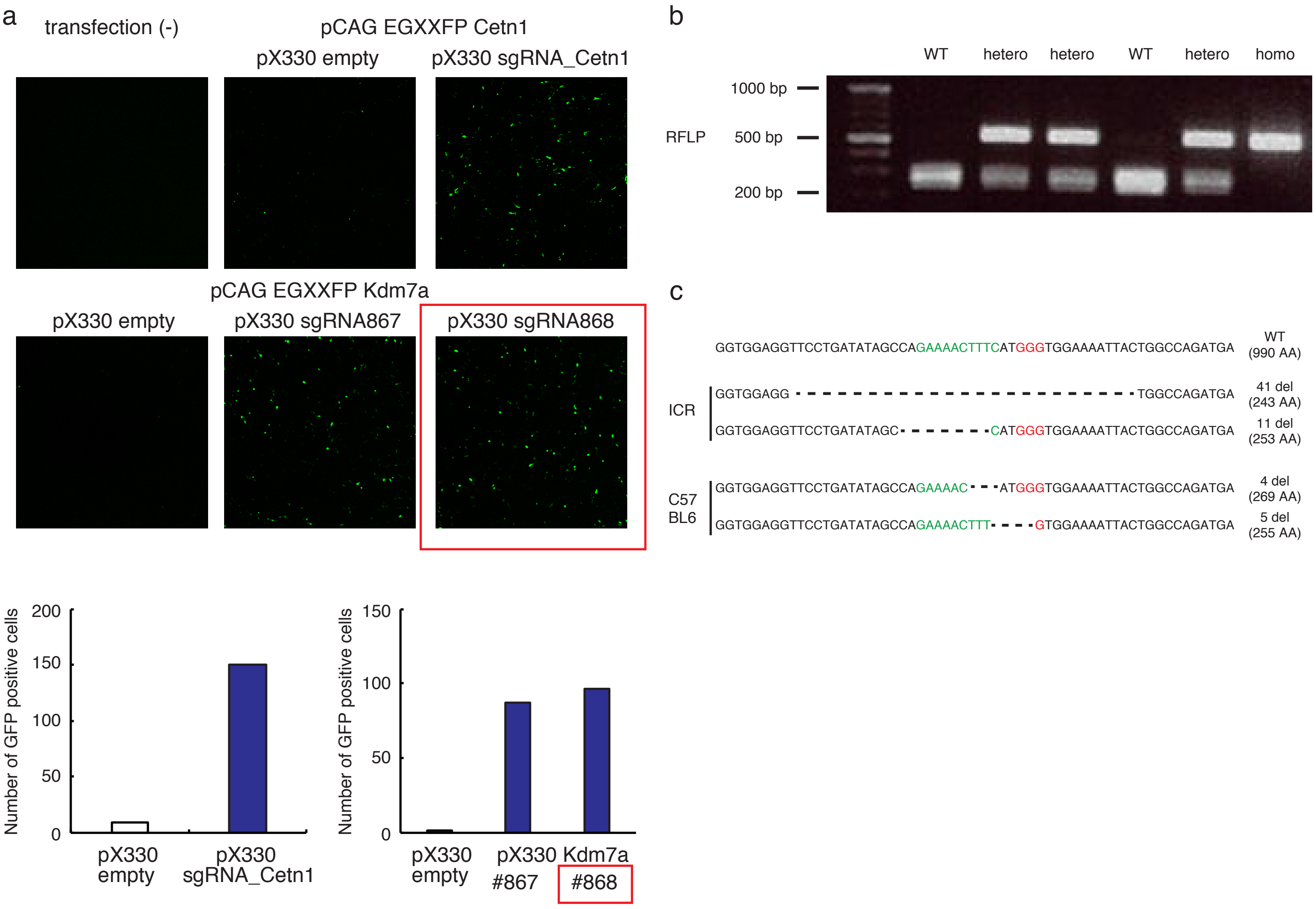

Supplementary Figure 1. Selection of an effective sgRNA for Kdm7a targeting.

(a) Validation of double-strand break (DSB)-mediated homology-dependent repair by reconstitution of the enhanced green fluorescent protein (EGFP). The approximately 500-bp genomic fragment containing the sgRNA target sequence (Cetn1 for positive control or Kdm7a for sgRNA validation) was inserted in pCAG - EGxxFP target plasmid1. The pX330 plasmid contains a humanized Cas9 expression cassette and an sgRNA expression cassette. The sgRNA targeting *Cetn1* or *Kdm7a* (Cetn1: sgRNA_Cetn1, Kdm7a: sgRNA867 or sgRNA868) was cloned into the pX330 plasmid. Both pCAG-EGxxFP and pX330 plasmid were co-transfected into the HeLa cells. When the target sequence was digested by sgRNA-guided Cas9 endonuclease, homology dependent repair (HR, homologous recombination; SSA, single-strand annealing) resulted in the reconstitution of the EGFP expression cassette. Bar plots showing the number of EGFP positive cells. (b) Genotyping of *Kdm7a* mutant mice by using restriction fragment length polymorphism (RFLP). *Kdm7a* PCR products were digested with PdmI. Representative RFLP result of C57BL/6 background is shown. (c) The sequence of mutant alleles in *Kdm7a* KO mice from ICR or C57BL/6 backgrounds. PAM sequence and the restriction site are labeled in red and green, respectively. All mutant mice are carrying frameshift mutation, and the number of deleted nucleotides and total amino acids (AA) is shown in the right column.

a

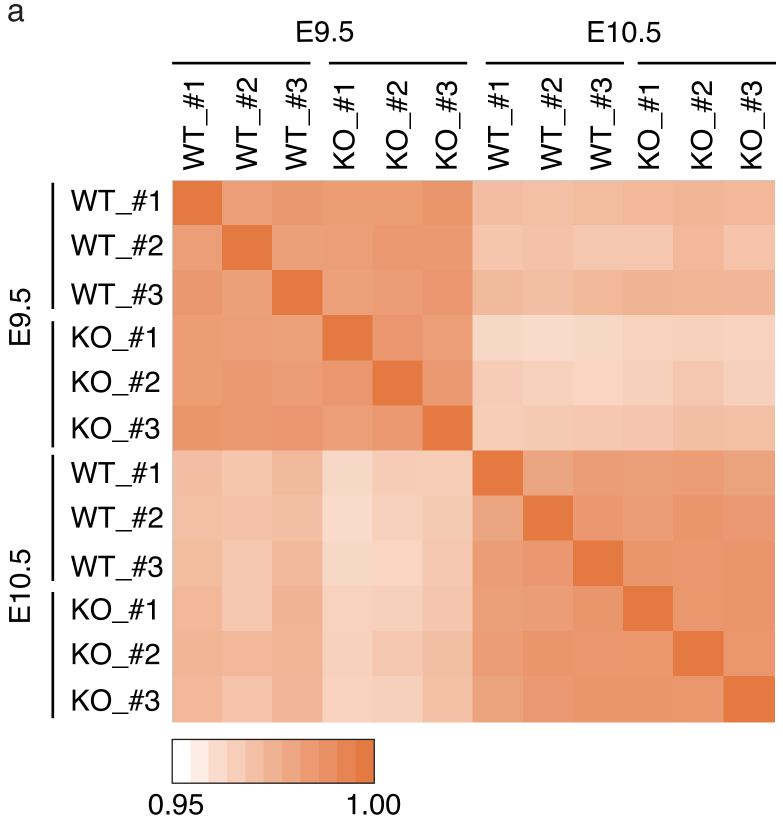

b

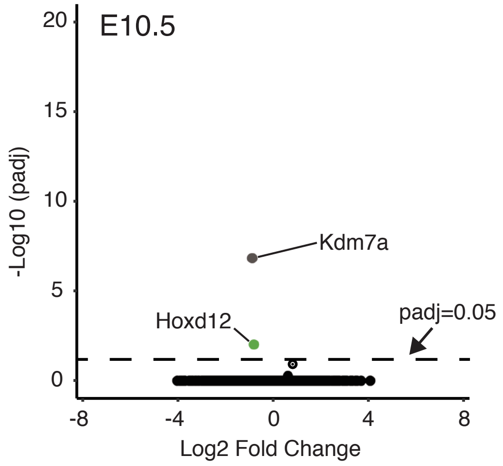

Supplementary Figure 2. Reproducibility of RNA-seq results.

(a) Scatter plots showing reproducibility of RNA-seq signals between the three biological replicates in Figure 2.

(b) Volcano plots showing differentially expressed genes in the wild-type and *Kdm7a*$^{-/-}$ embryos (n=3 for each genotype) at E10.5 The X- and Y-axes indicate the log2 fold-change and –log10 adjusted P-value (padj) produced by DESeq2, respectively.

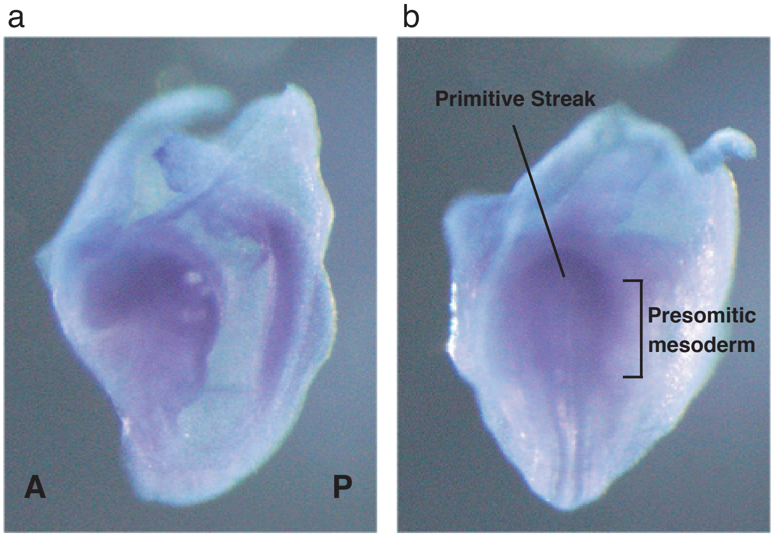

Supplementary Figure 3. Kdm7a RNA expression in embryos.

(a and b)Whole-mount in situ hybridization of Kdm7a mRNA in the wild type embryos at E8.5. Right lateral (a) and ventral (b) views of *Kdm7a*$^{-/-}$ mice are shown. The expression of Kdm7a was observed in primitive streak as well as presomitic mesoderm. A and P indicate anterior and posterior, respectively.

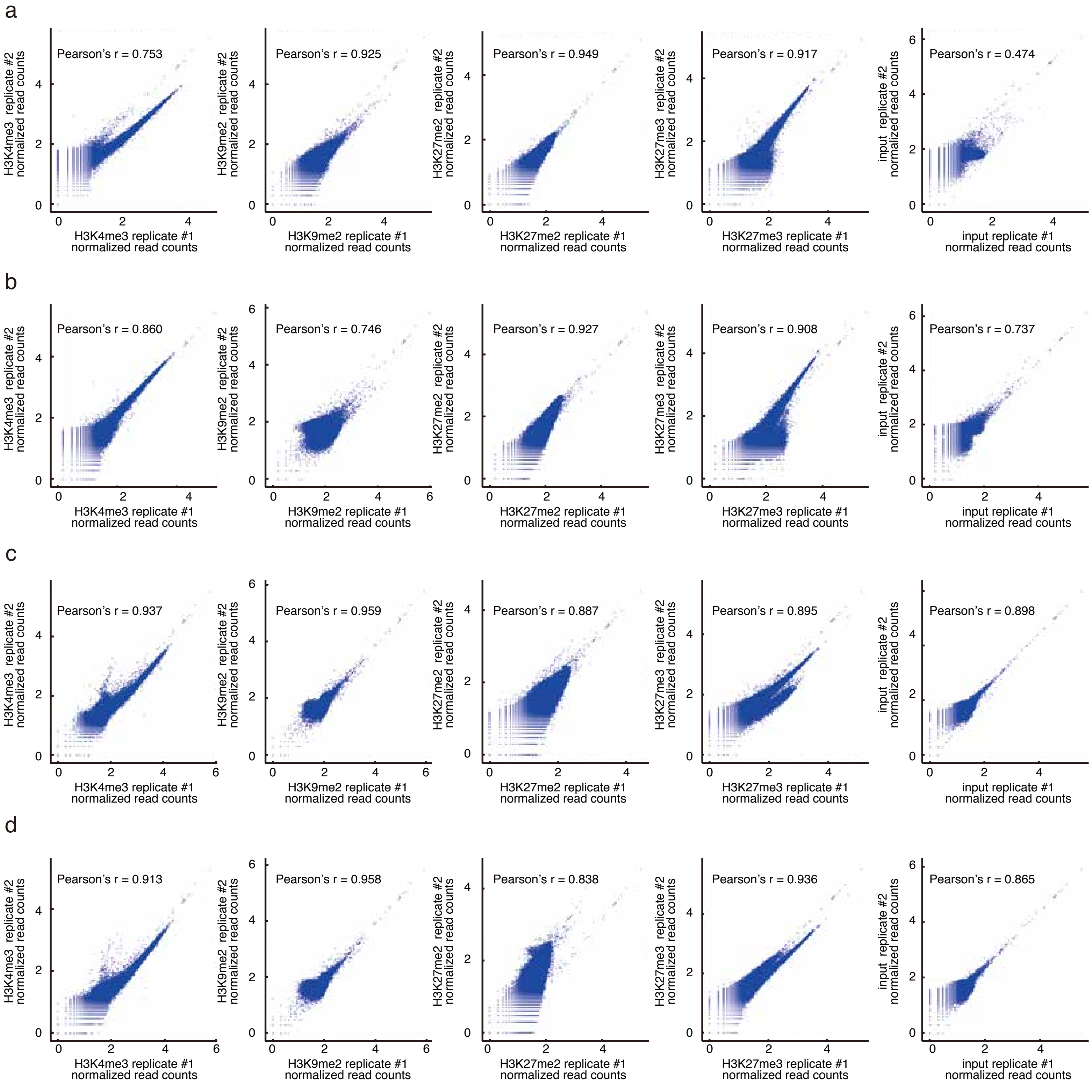

Supplementary Figure 4. Reproducibility of ChIP-Seq results.

(a-d) Scatter plots showing reproducibility of ChIP-Seq signals for H3K4me3, H3K9me2, H3K27me2, and H3K27me3 and correspondent input between the two biological replicates of trunk (a) and head (b) regions of the wild-type embryo, and trunk of wild-type (c) and *Kdm7a*$^{-/-}$ (d) embryos. Pearson correlation coefficient (Pearson's r) is described.

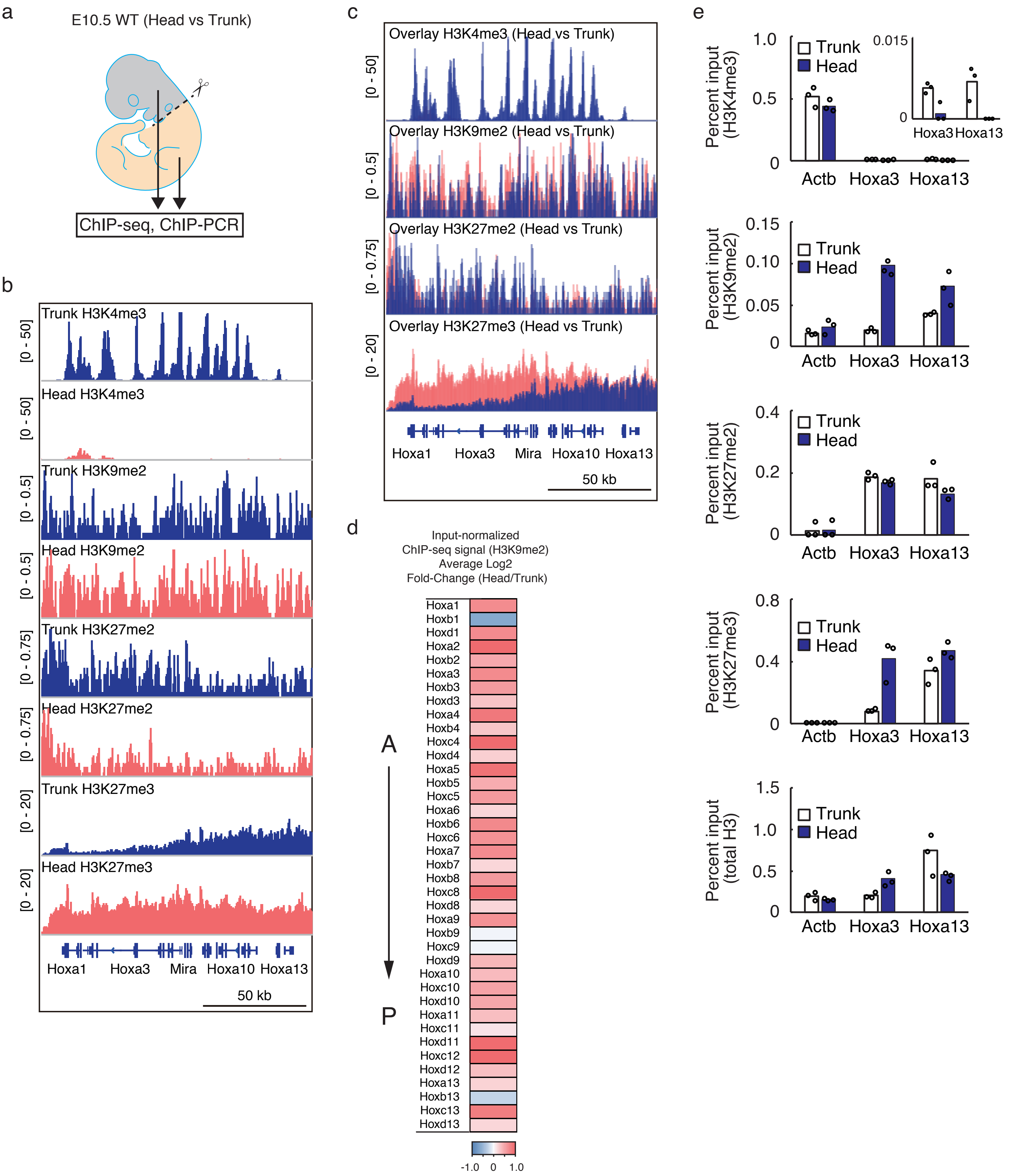
a
E10.5 WT (Head vs Trunk)
ChIP-seq, ChIP-PCR
b
Trunk H3K4me3
Head H3K4me3
Trunk H3K9me2
Head H3K9me2
Trunk H3K27me2
Head H3K27me2
Trunk H3K27me3
Head H3K27me3
[0 - 50]
[0 - 0.5]
[0 - 0.75]
[0 - 20]
Hoxa1 Hoxa3 Mira Hoxa10 Hoxa13
50 kb
c
Overlay H3K4me3 (Head vs Trunk)
Overlay H3K9me2 (Head vs Trunk)
Overlay H3K27me2 (Head vs Trunk)
Overlay H3K27me3 (Head vs Trunk)
Hoxa1 Hoxa3 Mira Hoxa10 Hoxa13
50 kb
d
Input-normalized
ChIP-seq signal (H3K9me2)
Average Log2
Fold-Change (Head/Trunk)
A
P
Hoxa1 Hoxb1 Hoxd1 Hoxa2 Hoxb2 Hoxa3 Hoxb3 Hoxd3 Hoxa4 Hoxb4 Hoxc4 Hoxd4 Hoxa5 Hoxb5 Hoxc5 Hoxa6 Hoxb6 Hoxc6 Hoxa7 Hoxb7 Hoxb8 Hoxc8 Hoxd8 Hoxa9 Hoxb9 Hoxc9 Hoxd9 Hoxa10 Hoxc10 Hoxd10 Hoxa11 Hoxc11 Hoxd11 Hoxc12 Hoxd12 Hoxa13 Hoxb13 Hoxc13 Hoxd13
-1.0 0 1.0
e
Percent input (H3K4me3)
Percent input (H3K9me2)
Percent input (H3K27me2)
Percent input (H3K27me3)
Percent input (total H3)
Trunk
Head
Actb Hoxa3 Hoxa13
0.015
Hoxa3 Hoxa13

Supplementary Figure 5. Differences of histone marks around Hoxa cluster between the developmental brain and somatic regions.

(a) The developmental brain and the posterior part of embryo (referred to as the “head” and “trunk” , respectively) from the wild-type embryo at E10.5 (colored grey and beige, respectively) were used for chromatin immunoprecipitation (ChIP)-Seq and ChIP-qPCR. (b and c) Gene tracks of ChIP-Seq signals for H3K4me3, H3K9me2, H3K27me2 and H3K27me3 close to the Hoxa cluster in the head and trunk regions of the wild-type embryo. ChIP-Seq signals were visualized by Integrative Genomics Viewer (http://software.broadinstitute.org/software/igv/) on the separate (b) and overlay (c) view.

(d) Heatmaps showing the average log2 fold-change of input-normalized H3K9me2 ChIP-Seq signals in the *Hox* genes between the head and trunk regions from the wild-type embryos. Red to blue coloring indicates the fold-change.

(e) ChIP-qPCR of H3K4me3, H3K9me2, H3K27me2, H3K27me3, and total H3 at the *Actb, Hoxa3, and Hoxa13* TSS in the head and trunk regions from the wild-type embryos, normalized to input. The data represent means from n = 3 technical replicates; independent experiments were repeated two or three times with similar results.

Supplementary References

1 Mashiko, D. et al. Generation of mutant mice by pronuclear injection of circular plasmid expressing Cas9 and single guided RNA. Scientific reports 3, 3355, doi:10.1038/srep03355 (2013).
