# Supplementary material for: Lysine demethylase 7a regulates murine anterior-posterior development by modulating the transcription of Hox gene cluster
Source: Commun Biol. 2020 Nov 30;3:725. doi: 10.1038/s42003-020-01456-5 (PMC7704666; doi:10.1038/s42003-020-01456-5)
Supplement: Supplementary file 3 — Description of Additional Supplementary Files [file 42003_2020_1456_MOESM3_ESM.pdf]

Description of Additional Supplementary Files

File Name: Supplementary Data 1
Description: Summary of RNA-Seq analysis, related to Figure 2

File Name: Supplementary Data 2
Description: RNA-Seq analysis for histone methyltransferases and demethylases

File Name: Supplementary Data 3
Description: Source data underlying the graphs presented in the main figures
