# Supplementary material for: Lysine demethylase 7a regulates murine anterior-posterior development by modulating the transcription of Hox gene cluster
Source: Commun Biol. 2020 Nov 30;3:725. doi: 10.1038/s42003-020-01456-5 (PMC7704666; doi:10.1038/s42003-020-01456-5)
Supplement: Supplementary file 7 — Reporting Summary [file 42003_2020_1456_MOESM7_ESM.pdf]

nature research

Corresponding author(s): Yasuharu Kanki

Last updated by author(s): Oct 12, 2020

Reporting Summary

Nature Research wishes to improve the reproducibility of the work that we publish. This form provides structure for consistency and transparency in reporting. For further information on Nature Research policies, see Authors & Referees and the Editorial Policy Checklist .

Statistics

For all statistical analyses, confirm that the following items are present in the figure legend, table legend, main text, or Methods section.

n/a	Confirmed	
☐	☒	The exact sample size (*n*) for each experimental group/condition, given as a discrete number and unit of measurement
☐	☒	A statement on whether measurements were taken from distinct samples or whether the same sample was measured repeatedly
☐	☒	The statistical test(s) used AND whether they are one- or two-sided<br>*Only common tests should be described solely by name; describe more complex techniques in the Methods section.*
☒	☐	A description of all covariates tested
☒	☐	A description of any assumptions or corrections, such as tests of normality and adjustment for multiple comparisons
☐	☒	A full description of the statistical parameters including central tendency (e.g. means) or other basic estimates (e.g. regression coefficient) AND variation (e.g. standard deviation) or associated estimates of uncertainty (e.g. confidence intervals)
☐	☒	For null hypothesis testing, the test statistic (e.g. *F*, *t*, *r*) with confidence intervals, effect sizes, degrees of freedom and *P* value noted<br>*Give P values as exact values whenever suitable.*
☒	☐	For Bayesian analysis, information on the choice of priors and Markov chain Monte Carlo settings
☒	☐	For hierarchical and complex designs, identification of the appropriate level for tests and full reporting of outcomes
☒	☐	Estimates of effect sizes (e.g. Cohen's *d*, Pearson's *r*), indicating how they were calculated

Our web collection on statistics for biologists contains articles on many of the points above.

Software and code

Policy information about availability of computer code

Data collection	The Illumina CASAVA1.8.2 software was used for basecalling and demultiplexing.
Data analysis	Software used in this study are as follows; Bowtie2 (http://bowtie-bio.sourceforge.net/bowtie2/index.shtml), FastQC (http://www.bioinformatics.babraham. ac.uk/projects/fastqc), HISAT2 (https://ccb.jhu.edu/software/hisat2/index.shtml), StringTie (https://ccb.jhu.edu/software/stringtie/), deepTools (https://deeptools.readthedocs.io/en/develop/), featureCounts (http://bioinf.wehi.edu.au/featureCounts/), R-package: DESeq2 (https://bioconductor.org/packages/release/bioc/html/DESeq2.html), DAVID (https://david.ncifcrf.gov), Ingenuity Pathway Analysis (https://www.qiagenbioinformatics.com/products/ingenuity-pathway-analysis/), Integrative Genomics Viewer (http://software.broadinstitute.org/software/igv/), Trimmomatic (http://www.usadellab.org/cms/?page=trimmomatic), SAMtools (http://www.htslib.org).

For manuscripts utilizing custom algorithms or software that are central to the research but not yet described in published literature, software must be made available to editors/reviewers. We strongly encourage code deposition in a community repository (e.g. GitHub). See the Nature Research guidelines for submitting code & software for further information.

Data

Policy information about availability of data

All manuscripts must include a data availability statement. This statement should provide the following information, where applicable:

- Accession codes, unique identifiers, or web links for publicly available datasets
- A list of figures that have associated raw data
- A description of any restrictions on data availability

Data generated for this study has been deposited to the NCBI Gene Expression Omnibus (GEO) under the accession number GSE133188.

Field-specific reporting

Please select the one below that is the best fit for your research. If you are not sure, read the appropriate sections before making your selection.

☒ Life sciences ☐ Behavioural & social sciences ☐ Ecological, evolutionary & environmental sciences

For a reference copy of the document with all sections, see nature.com/documents/nr-reporting-summary-flat.pdf

Life sciences study design

All studies must disclose on these points even when the disclosure is negative.

Sample size	Experiments were conducted with cell lines or laboratory animals with multiple available biological replicates and based on previous experience with specific experimental setup.
Data exclusions	N/A
Replication	In each biological experiment, at least two or three independent repeats were performed.<br>RNA-seq and ChIP-seq experiments were done with three and two biological replicates, respectively, and each reproducibility was confirmed by correlation coefficients.
Randomization	Randomization was not relevant to this study.
Blinding	Blinding was not possible for this study.

Reporting for specific materials, systems and methods

We require information from authors about some types of materials, experimental systems and methods used in many studies. Here, indicate whether each material, system or method listed is relevant to your study. If you are not sure if a list item applies to your research, read the appropriate section before selecting a response.

Materials & experimental systems

n/a	Involved in the study	
☐	☒	Antibodies
☐	☒	Eukaryotic cell lines
☒	☐	Palaeontology
☐	☒	Animals and other organisms
☒	☐	Human research participants
☒	☐	Clinical data

Methods

n/a	Involved in the study	
☐	☒	ChIP-seq
☒	☐	Flow cytometry
☒	☐	MRI-based neuroimaging

Antibodies

Antibodies used	H3K4me3 MAB Institute Cat# MABI0304; RRID:AB_11123891<br>H3K9me2 MAB Institute Cat#MABI0307; RRID:AB_11124951<br>H3K27me2 Cell Signaling Technology Cat#9728; AB_1281338<br>H3K27me3 MAB Institute Cat#MABI0307; MABI0323<br>total H3 Abcam Cat#ab1791; AB_302613
Validation	Certified and company-validated antibodies were purchased and used in this study.

Eukaryotic cell lines

Policy information about cell lines

Cell line source(s)	HeLa; ATCC
Authentication	Human cervical cancer cell line, HeLa, was purchased from ATCC (Manassas, VA) and grown and passaged every 2 or 3 days in DMEM (nacalai tesque, Kyoto, Japan), supplemented with 1% penicillin/streptomycin (Wako, Osaka, Japan) and 10% FBS (Thermo Fisher Scientific, Waltham, MA). The cells were cultured at 37 ºC and in a 5% CO2 atmosphere in a humidified incubator.
Mycoplasma contamination	Cell lines were routinely tested for mycoplasma contamination.

Commonly misidentified lines
(See ICLAC register)

No commonly misidentified cell lines were used in this study.

Animals and other organisms

Policy information about studies involving animals; ARRIVE guidelines recommended for reporting animal research

Laboratory animals	C57BL/6N mice (from Japan SLC), ICR mice (from Charles River Laboratories)
Wild animals	No wild animals were used for this study.
Field-collected samples	No field collected samples were used for this study.
Ethics oversight	All mouse experiments were approved by The University of Tokyo Animal Care and Use Committee (approval number H28-1).

Note that full information on the approval of the study protocol must also be provided in the manuscript.

ChIP-seq

Data deposition

- [x] Confirm that both raw and final processed data have been deposited in a public database such as GEO.
- [x] Confirm that you have deposited or provided access to graph files (e.g. BED files) for the called peaks.

Data access links
May remain private before publication.

https://www.ncbi.nlm.nih.gov/geo/query/acc.cgi?acc=GSE133188

Files in database submission

brain_input_rep1.fastq.gz
somite_input_rep1.fastq.gz
brain_H3K4me3_rep1.fastq.gz
somite_H3K4me3_rep1.fastq.gz
brain_H3K9me2_rep1.fastq.gz
somite_H3K9me2_rep1.fastq.gz
wt_input_rep1.fastq.gz
ko_input_rep1.fastq.gz
wt_H3K4me3_rep1.fastq.gz
ko_H3K4me3_rep1.fastq.gz
wt_H3K9me2_rep1.fastq.gz
ko_H3K9me2_rep1.fastq.gz
wt_input_rep2.fastq.gz
ko_input_rep2.fastq.gz
brain_input_rep2.fastq.gz
somite_input_rep2.fastq.gz
wt_H3K4me3_rep2.fastq.gz
ko_H3K4me3_rep2.fastq.gz
wt_H3K9me2_rep2.fastq.gz
ko_H3K9me2_rep2.fastq.gz
brain_H3K4me3_rep2.fastq.gz
somite_H3K4me3_rep2.fastq.gz
brain_H3K9me2_rep2.fastq.gz
somite_H3K9me2_rep2.fastq.gz
somite_H3K27_input_rep1.fastq.gz
somite_H3K27me3_rep1.fastq.gz
somite_H3K27me2_rep1.fastq.gz
brain_H3K27_input_rep1.fastq.gz
brain_H3K27me3_rep1.fastq.gz
brain_H3K27me2_rep1.fastq.gz
somite_H3K27me3_rep2.fastq.gz
somite_H3K27me2_rep2.fastq.gz
brain_H3K27me3_rep2.fastq.gz
brain_H3K27me2_rep2.fastq.gz
wt_H3K27_input_rep3.fastq.gz
wt_H3K27me3_rep3.fastq.gz
wt_H3K27me2_rep3.fastq.gz
ko_H3K27_input_rep3.fastq.gz
ko_H3K27me3_rep3.fastq.gz
ko_H3K27me2_rep3.fastq.gz
wt_H3K27_input_rep4.fastq.gz

wt_H3K27me3_rep4.fastq.gz
wt_H3K27me2_rep4.fastq.gz
ko_H3K27_input_rep4.fastq.gz
ko_H3K27me3_rep4.fastq.gz
ko_H3K27me2_rep4.fastq.gz

Genome browser session
(e.g. UCSC)

Provide a link to an anonymized genome browser session for "Initial submission" and "Revised version" documents only, to enable peer review. Write "no longer applicable" for "Final submission" documents.

Methodology

Replicates

ChIP-seq experiments were performed by using a single biological sample per each experimental condition. ChIP-seq results were confirmed by ChIP-qPCR experiments with at least two biological replicates.

Sequencing depth

All experiments were sequenced using single-end sequencing with 50 basepair reads. Sequencing depth information is as follows:
Sample, Total reads, Mapped reads, Uniquely mapped reads
brain_input_rep1 7,154,905 6,226,652 6,150,865
somite_input_rep1 3,559,514 2,345,254 2,254,614
brain_H3K4me3_rep1 14,513,137 8,897,757 8,691,215
somite_H3K4me3_rep1 14,735,155 7,050,170 6,495,419
brain_H3K9me2_rep1 23,561,851 22,389,357 22,101,375
somite_H3K9me2_rep1 20,546,837 18,992,200 18,714,420
wt_input_rep1 7,440,437 6,986,249 6,927,072
ko_input_rep1 6,312,976 5,591,368 5,541,722
wt_H3K4me3_rep1 38,846,012 34,780,168 34,265,146
ko_H3K4me3_rep1 35,233,544 31,471,160 30,923,088
wt_H3K9me2_rep1 34,552,454 31,170,240 30,904,646
ko_H3K9me2_rep1 31,660,448 27,638,793 27,412,699
wt_input_rep2 20,543,351 19,047,164 18,764,793
ko_input_rep2 16,733,195 15,658,837 15,419,580
brain_input_rep2 27,186,465 24,593,236 24,174,781
somite_input_rep2 23,492,081 20,874,890 20,503,342
wt_H3K4me3_rep2 21,054,575 18,610,983 13,327,505
ko_H3K4me3_rep2 25,216,015 14,731,816 8,179,809
wt_H3K9me2_rep2 25,163,969 24,265,070 22,531,175
ko_H3K9me2_rep2 26,809,631 24,633,443 22,599,588
brain_H3K4me3_rep2 30,145,232 20,648,903 18,725,706
somite_H3K4me3_rep2 50,953,030 22,723,011 17,166,914
brain_H3K9me2_rep2 27,018,743 24,218,440 22,354,125
somite_H3K9me2_rep2 17,161,724 15,844,439 14,408,271
somite_H3K27_input_rep1 33,261,414 28,932,926 28,264,229
somite_H3K27me3_rep1 18,842,860 12,927,452 12,427,733
somite_H3K27me2_rep1 20,870,557 16,038,920 15,100,181
brain_H3K27_input_rep1 26,091,438 20,692,720 20,154,090
brain_H3K27me3_rep1 21,831,743 20,443,243 20,086,537
brain_H3K27me2_rep1 24,276,417 19,634,662 18,633,128
somite_H3K27me3_rep2 22,361,316 16,698,746 15,117,135
somite_H3K27me2_rep2 16,800,621 9,951,183 9,537,056
brain_H3K27me3_rep2 24,482,001 17,781,918 17,347,509
brain_H3K27me2_rep2 21,792,082 19,613,054 18,831,516
wt_H3K27_input_rep3 14,255,864 9,422,187 8,710,797
wt_H3K27me3_rep3 16,443,819 11,564,305 11,157,045
wt_H3K27me2_rep3 14,125,220 10,611,649 9,913,547
ko_H3K27_input_rep3 16,475,195 12,732,566 11,946,886
ko_H3K27me3_rep3 18,888,228 13,022,839 12,562,884
ko_H3K27me2_rep3 16,986,397 14,198,103 13,206,054
wt_H3K27_input_rep4 26,286,509 25,505,739 25,058,546
wt_H3K27me3_rep4 18,243,305 14,399,471 14,071,945
wt_H3K27me2_rep4 13,797,983 10,663,163 8,537,896
ko_H3K27_input_rep4 24,931,735 19,323,734 18,673,221
ko_H3K27me3_rep4 21,601,508 17,070,059 16,662,503
ko_H3K27me2_rep4 14,358,423 12,597,462 11,407,493

Antibodies

H3K4me3 MAB Institute Cat# MABI0304; RRID:AB_11123891
H3K9me2 MAB Institute Cat#MABI0307; RRID:AB_11124951

Peak calling parameters	Sequence reads were trimmed using Trimmomatic with 'ILLUMINACLIP:adaptor_sequence.fa:2:30:7 LEADING:3 TRAILING:3 SLIDINGWINDOW:4:15 MINLEN:36' parameters. The trimmed reads were aligned to the mouse reference genome mm10 using Bowtie2 with default parameters. SAM files were sorted and converted into BAM files using Samtools.
Data quality	The quality of FASTQ files was assessed using FastQC. Low quality bases and adaptor sequences were trimmed using Trimmomatic as described above.
Software	FastQC 0.11.8 – Sequence quality check<br>Trimmomatic 0.38 – Read trimming<br>Bowtie2 2.3.4.3 – Read mapping<br>Samtools 1.9 – Sorting and converting SAM files into BAM files<br>deepTools 3.2.0 – Generating BIGWIG files<br>featureCounts – Generating read count matrices
